# Non-contiguous finished genome sequence and description of *Holdemania massiliensis* sp. nov.

**DOI:** 10.4056/sigs.4628316

**Published:** 2013-12-15

**Authors:** Ajay Kumar Mishra, Jean-Christophe Lagier, Anne Pfleiderer, Thi Thien Nguyen, Aurelia Caputo, Didier Raoult, Pierre-Edouard Fournier

**Affiliations:** 1 URMITE, CNRS 7278, IRD 198, Inserm U1095, Aix-Marseille Université, Faculté de Médecine, Marseille, France*; 2 King Fahad Medical Research Center, King Abdul Aziz University, Jeddah, Saudi Arabia

**Keywords:** *Holdemania massiliensis*, genome, culturomics, taxono-genomics

## Abstract

*Holdemania massiliensis* strain AP2^T^ sp. nov. is the type strain of *H. massiliensis* sp. nov., a new species within the genus *Holdemania*. This strain, whose genome is described here, was isolated from the fecal flora of a 21-year-old French Caucasian female suffering from severe restrictive anorexia nervosa. *H. massiliensis* is a Gram-positive, anaerobic bacillus. Here we describe the features of this organism, together with the complete genome sequence and annotation. The 3,795,625 bp-long genome (one chromosome but no plasmid) contains 3,461 protein-coding and 49 RNA genes, including 3 rRNA genes.

## Introduction

*Holdemania massiliensis* strain AP2^T^ (= CSUR P195 = DSM 26143) is the type strain of *H. massiliensis* sp. nov. This bacterium is a Gram-positive, non-spore-forming, indole negative, anaerobic and non-motile bacillus that was isolated from the stool of a 21-year-old woman suffering from anorexia as part of a “culturomics” study aiming to individually cultivate all species within human feces [1-3].

The current prokaryotic species classification, known as polyphasic taxonomy, is based on a combination of genomic and phenotypic properties [4]. The number of sequenced genomes is increasing exponentially and in parallel with the decreasing cost of sequencing. To date, more than 6,000 bacterial genomes have been published and approximately 25,000 genomes project are anticipated to be completed in a near future [5]. We recently proposed to integrate genomic information in the taxonomic framework and description of new bacterial species [6-27].

Here we present a summary classification and a set of features for *H. massiliensis* sp. nov. strain AP2^T^ (= CSUR P195 = DSM 26143), together with the description of the complete genomic sequencing and annotation. These characteristics support the circumscription of the species *H. massiliensis*. The genus *Holdemania* (Willems *et al*. 1997) was created in 1997 on the basis of 16S rDNA gene sequence, biochemical tests, fatty acid and cell wall murein analysis [28]. To date, this genus includes a single species, *H. filiformis*, which was isolated from feces of healthy humans [29].

## Classification and features

A stool sample was collected from a 21-year-old French Caucasian female suffering from severe restrictive anorexia nervosa, who had been hospitalized for recurrent weight loss and aggravation of her general state. She had an eight year history of mental anorexia. The patient gave an informed and signed consent. Both this study and the assent procedure were approved by the Ethics Committee of the Institut Fédératif de Recherche IFR48, Faculty of Medicine, Marseille, France and the agreement of the ethics committee of the IFR48 (Marseille, France) was obtained under reference 09-022. Several other new bacterial species were isolated from diverse stool samples using microbial culturomics [6-27]. The fecal specimen from the patient was preserved at -80°C immediately after collection. Strain AP2^T^ (Table 1) was isolated in November 2011 after preincubation in an anaerobic blood culture bottle with the addition of 5ml of thioglycolate, and inoculation in Columbia agar (BioMerieux, Marcy l’Etoile, France).

**Table 1:** Classification and general features of *Holdemania massiliensis* strain AP2^T^ according to the MIGS recommendations [30]

**MIGS ID**	**Property**	**Term**	**Evidence code^a^**
	Current classification	Domain *Bacteria* Phylum *Firmicutes* Class *Erysipelotrichia* Order *Erysipelotrichales* Family *Erysipelotrichaceae* Genus *Holdemania* Species *Holdemania massiliensis* Type strain AP2^T^	TAS [31] TAS [32-34] TAS [35,36] TAS [36,37] TAS [38] TAS [28] IDA IDA
	Gram stain	positive	IDA
	Cell shape	Bacillus	IDA
	Motility	Not motile	IDA
	Sporulation	Non-sporulating	IDA
	Temperature range	Mesophile	IDA
	Optimum temperature	37°C	IDA
MIGS-6.3	Salinity	Unknown	IDA
MIGS-22	Oxygen requirement	Anaerobic	IDA
	Carbon source	Unknown	NAS
	Energy source	Unknown	NAS
MIGS-6	Habitat	Human gut	IDA
MIGS-15	Biotic relationship	Free living	IDA
MIGS-14	Pathogenicity Biosafety level Isolation	Unknown 2 Human feces	NAS
MIGS-4	Geographic location	France	IDA
MIGS-5	Sample collection time	November 2011	IDA
MIGS-4.1	Latitude Longitude	43.296482 5.36978	IDA IDA
MIGS-4.3	Depth	Surface	IDA
MIGS-4.4	Altitude	0 m above sea level	IDA

Evidence codes - IDA: Inferred from Direct Assay; TAS: Traceable Author Statement (i.e., a direct report exists in the literature); NAS: Non-traceable Author Statement (i.e., not directly observed for the living, isolated sample, but based on a generally accepted property for the species, or anecdotal evidence). These evidence codes are from the Gene Ontology project [39]. If the evidence is IDA, then the property was directly observed for a live isolate by one of the authors or an expert mentioned in the acknowledgements.

**Figure 1:**
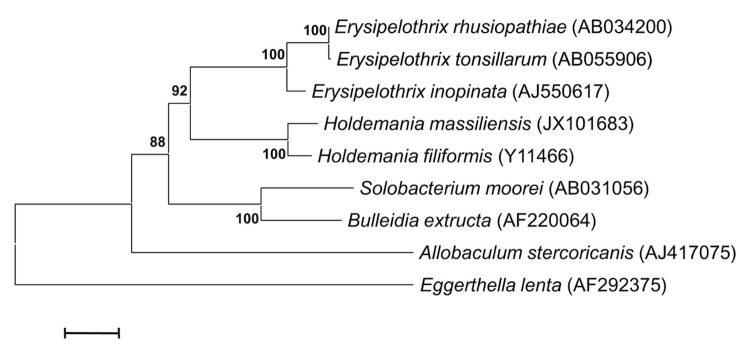
Phylogenetic tree highlighting the position of *Holdemania massiliensis* strain AP2^T^ relative to other type strains within the *Erysipelotrichaceae* family. GenBank accession numbers are indicated in parentheses. Sequences were aligned using CLUSTALW, and phylogenetic inferences obtained using the maximum-likelihood method within MEGA program. Numbers at the nodes are percentages of bootstrap values obtained by repeating the analysis 500 times to generate a majority consensus tree. *Eggerthella lenta* was used as outgroup. The scale bar represents a 2% nucleotide sequence divergence.

This strain exhibited a 97% nucleotide sequence similarity with *H. filiformis* [28], and a range of 90-91% nucleotide sequence similarity to most closely related members of the genus *Erysipelothrix* [29] (Figure 1). This value was lower than the 98.7% 16S rRNA gene sequence threshold recommended by Stackebrandt and Ebers to delineate a new species without carrying out DNA-DNA hybridization [40].

**Figure 2:**
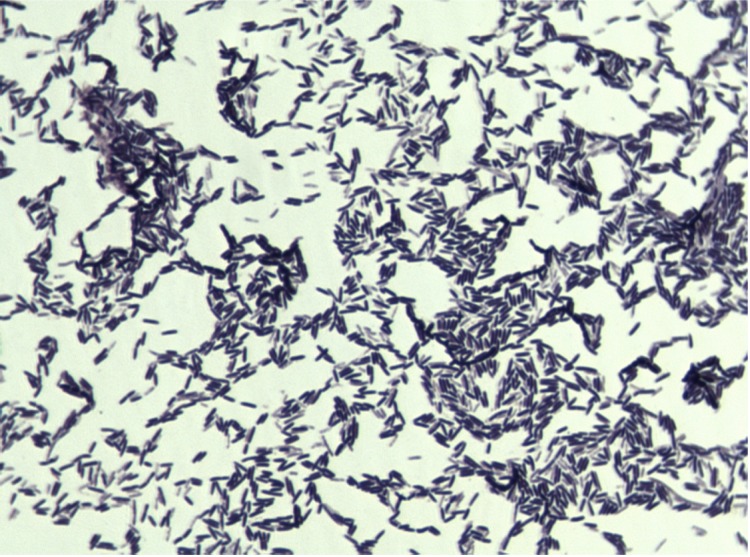
Gram staining of *H. massiliensis* strain AP2^T^

**Figure 3:**
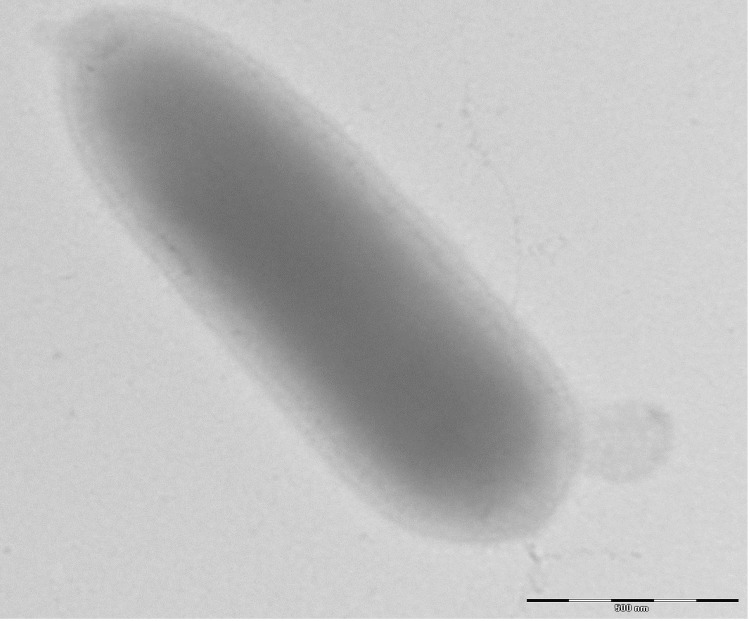
Transmission electron microscopy of *H. massiliensis* strain AP2^T^, using a Morgani 268D (Philips) at an operating voltage of 60kV. The scale bar represents 500 nm.

Different growth temperatures (25, 30, 37, 45°C) were tested. Growth occurred at 25 and 30°C after 72 hours of inoculation and the optimal growth was observed at 37°C after 24 hours of inoculation. Colonies were 0.2 mm in diameter, light grey, with α-haemolysis on blood-enriched Columbia agar. Growth of the strain was tested under anaerobic and microaerophilic conditions using GENbag anaer and GENbag microaer systems, respectively (BioMérieux), and under aerobic conditions with or without 5% CO_2_. The strain growth was obtained only in anaerobic condition. The motility test was negative. Cells grown on agar are Gram-positive rods (Figure 2). The mean dimensions by electron microscopy were 0.57 µm in width and 1.75 µm in length (Figure 3).

Strain AP2^T^ exhibited a positive oxidase but no catalase activity. Using an API rapid 32A strip (Biomerieux), positive reactions were obtained for β-galactosidase, α-glucosidase, α-fucosidase and pyroglutamic acid arylamidase. Substrate oxidation and assimilation was examined using an API 50CH strip (Biomerieux) at 37°C. Positive reactions were observed for glycerol, D-ribose, D-galactose, D-glucose, D-fructose, D-mannose, inositol, D-mannitol, D-sorbitol, N-acetyl-glucosamine, amygdalin, arbutin, esculin, salicin, D-cellobiose, D-maltose, D-lactose, D-saccharose, D-melezitose, gentiobiose, D-tagatose and postassium gluconate. *H. massiliensis* is susceptible to amoxicillin, metronidazole, vancomycin, clindamycin and imipenem. The phenotypic characteristics that differentiate *H. massiliensis* from other species are summarized in Table 2.

**Table 2:** Differential characteristics of *Holdemania massiliensis* strain AP2^T^, *Holdemania filiformis* strain ATCC 51649, *Solobacterium moorei* strain CCUG 39336 and *Erysipelothrix rhusiopathiae* strain ATCC 19414^T^.

**Properties**	*H. massiliensis*	*H. filiformis*	*S. moorei*	*E. rhusiopathiae*
Cell diameter (µm)	0.57	1.0-1.2	0.8-1.4	0.8-2.5
Oxygen requirement	anaerobic	anaerobic	anaerobic	facultative anaerobes
Pigment production	+	–	–	–
Gram stain	+	+	+	+
Salt requirement	–	–	–	–
Motility	–	–	–	–
Peptidoglycan type	na	B1δ (L-Ala)-D-Glu-L-Asp-L-Lys-	na	B1δ (L-Ala)-D-Glu-Gly-L-Lys
Endospore formation	–	–	–	–
				
**Production of**				
Acid phosphatase		na	na	+
Catalase	+	–	na	–
Oxidase	–	na	na	–
Nitrate reductase	–	–	–	–
Urease	–	na	na	na
β-galactosidase	+	na	na	–
N-acetyl-glucosamine	–	na	na	+
				
**Acid from**				
L-Arabinose	–	w	–	–
Ribose	+	w	+	–
Mannose	+	w	–	w
Mannitol	+	w	–	–
Sucrose	–	+	–	w/-
D-glucose	+	+	+	+
D-fructose	+	+	+	+
D-maltose	+	w	+	+
D-lactose	+	w	–	+
				
**Hydrolysis of gelatin**	na	–	–	+
G+C content (mol%)	47.1	50.2	36.8	36.6
Habitat	human gut	human gut	human gut	animal feces, environmental, human infection

na = data not available; w = weak

**Figure 4:**
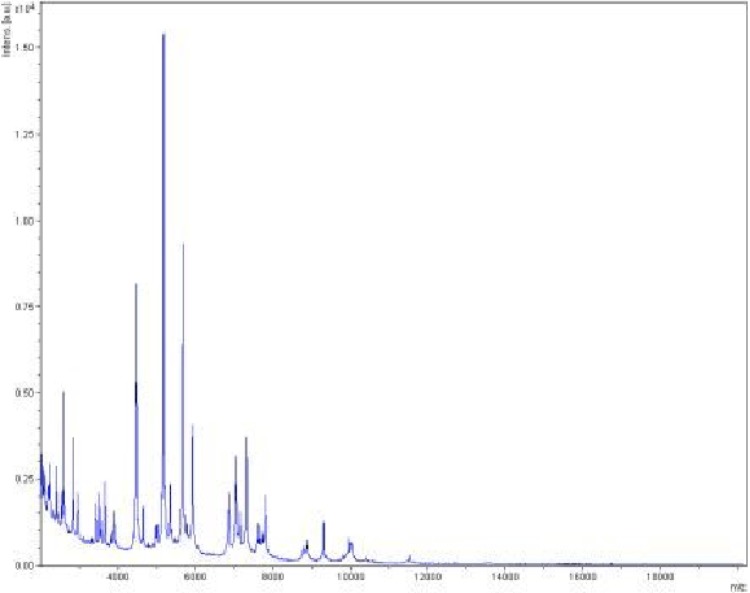
Reference mass spectrum from *H. massiliensis* strain AP2^T^. Spectra from 12 individual colonies were compared and a reference spectrum was generated.

**Figure 5:**
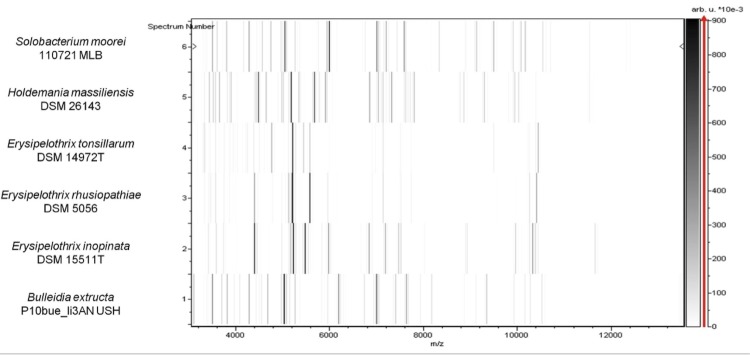
Gel view comparing *Holdemania massiliensis* strain AP2^T^, *Solobacterium moorei, Erysipelothrix tonsillarum, E. rhusiopathiae, E. inopinata* and *Bulleida exctructa.* The gel view displays the raw spectra of loaded spectrum files arranged in a pseudo-gel like look. The x-axis records the m/z value. The left y-axis displays the running spectrum number originating from subsequent spectra loading. The peak intensity is expressed as a gray scale. The color bar and the right y-axis indicate the relation between the color with which a peak is displayed and the peak intensity in arbitrary units. Displayed species are indicated on the left.

Matrix-assisted laser-desorption/ionization time-of-flight (MALDI-TOF) MS protein analysis was carried out as previously described [41] using a Microflex spectrometer (Bruker Daltonics, Leipzig, Germany). Twelve individual colonies were deposited on a MTP 384 MALDI-TOF target plate (Bruker). The twelve AP2^T^ spectra were imported into the MALDI BioTyper software (version 2.0, Bruker) and analyzed by standard pattern matching (with default parameter settings) against the main spectra of 4,706 bacteria. A score enabled the presumptive identification and discrimination of the tested species from those in a database: a score > 2 with a validated species enabled the identification at the species level; and a score < 1.7 did not enable any identification. For strain AP2^T^, no significant score was obtained, suggesting that our isolate was not a member of any known species (Figures 4 and 5). We added the spectrum from strain AP2^T^ to our database (Figure 4).

## Genome sequencing information

### Genome project history

The organism was selected for sequencing on the basis of its phylogenetic position and 16S rRNA similarity to other members of the *Holdemania* genus, and is part of a “culturomics” study of the human digestive flora aiming at isolating all bacterial species within human feces. It was the second draft genome of a *Holdemania* species and the first genome of *Holdemania massiliensis* sp. nov. A summary of the project information is shown in Table 3. The Genbank accession number is CALK00000000 and consists of 66 contigs. Table 3 shows the project information and its association with MIGS version 2.0 compliance [30].

**Table 3:** Project information

**MIGS ID**	**Property**	**Term**
MIGS-31	Finishing quality	High-quality draft
MIGS-28	Libraries used	One 454 paired end 3-kb library
MIGS-29	Sequencing platforms	454 GS FLX Titanium
MIGS-31.2	Fold coverage	54.25x
MIGS-30	Assemblers	Newbler version 2.5.3
MIGS-32	Gene calling method	Prodigal
	Gold ID	Gi20761
	INSDC ID	PRJEB187
	Genbank ID	CALK00000000
	Genbank Date of Release	July 31, 2012
	Project relevance	Study of the human gut microbiome

### Growth conditions and DNA isolation

*H. massiliensis* sp. nov. strain AP2^T^, (= CSUR P195= DSM 26143), was grown anaerobically on Columbia agar medium at 37°C. Five Petri dishes were spread and resuspended in 3x100µl of G2 buffer (EZ1 DNA Tissue kit, Qiagen). A first mechanical lysis was performed using glass powder in the Fastprep-24 device (Sample Preparation system, MP Biomedicals, USA) for 2×20 seconds. DNA was treated with 2.5 µg/µL of lysozyme (30 minutes at 37°C) and extracted using the BioRobot EZ 1 Advanced XL (Qiagen). The DNA was concentrated and purified on a Qiamp kit (Qiagen). The yield and the concentration of DNA was 69.3 ng/µl as measured by using Quant-it Picogreen kit (Invitrogen) on the Genios Tecan fluorometer

### Genome sequencing and assembly

DNA (5 µg) was mechanically fragmented for the paired-end sequencing, using a Covaris device (Covaris Inc., Woburn, MA,USA) with an enrichment size of 3-4 kb. The DNA fragmentation was visualized through an Agilent 2100 BioAnalyzer on a DNA Labchip 7500 which yielded an optimal size of 3.4 kb. The library was constructed using the 454 GS FLX Titanium paired-end rapid library protocol (Roche, Meylan, France). Circularization and nebulization were performed which generated a pattern of optimal size of 589 bp. PCR amplification was performed for 17 cycles followed by double size selection. The single-stranded paired-end library was quantified using a Quant-it Ribogreen Kit (Invitrogen) using the Genios Tecan fluorometer. The library concentration equivalence was calculated as 1.42× 10^10^ molecules/µL. The library was stored at -20°C until further use.

For the shotgun sequencing, DNA (500 ng) was mechanically fragmented using a Covaris device (Covaris Inc.) as described by the manufacturer. The DNA fragmentation was visualized using an Agilent 2100 BioAnalyzer on a DNA Labchip 7500 which yielded an optimal size of 1.7 kb. The library was constructed using the GS Rapid library Prep kit (Roche) and quantified using a TBS 380 mini fluorometer (Turner Biosystems, Sunnyvale, CA, USA). The library concentration equivalence was calculated as 2.8× 10^9^ molecules/µL. The library was stored at -20°C until further use.

The shotgun library was clonally amplified with 1 and 2 cpb in two emPCR reactions each, and the paired-end library was amplified with 0.5 cpb in three emPCR reactions using the GS Titanium SV emPCR Kit (Lib-L) v2 (Roche). The yields of the emPCR were 6.8 and 9.8%, respectively, for the shotgun library, and 11.29% for the paired-end library. These yields fall into the expected 5 to 20% range according to Roche protocol.

For each library, approximately 790,000 beads for a quarter region were loaded on the GS Titanium PicoTiterPlate PTP kit and sequenced with the GS FLX Titanium Sequencing Kit XLR70 (Roche). The run was performed overnight and analyzed on a cluster using the gsRunBrowser and Newbler assembler (Roche). For the shotgun sequencing, 188,659 passed-filter wells were obtained. The sequencing generated 129.3 Mb with an average length of 685 bp. For the paired-end sequencing, 106,675 passed-filter wells were obtained. The sequencing generated 35 Mb with an average length of 262 bp. The passed-filter sequences were assembled using Newbler with 90% identity and 40 bp as overlap. The final assembly identified 8 scaffolds and 66 contigs (>1,500 bp) and generated a genome size of 3.79 Mb which corresponds to a coverage of 54.25 genome equivalents.

### Genome annotation

Open Reading Frames (ORFs) were predicted using Prodigal [42] with default parameters, but the predicted ORFs were excluded if they were spanning a sequencing gap region. The predicted bacterial protein sequences were searched against the GenBank database [43] and the Clusters of Orthologous Groups (COG) databases using BLASTP. The tRNAScanSE tool [44] was used to find tRNA genes, whereas ribosomal RNAs were found by using RNAmmer [45] and BLASTn against the GenBank database. Lipoprotein signal peptides and numbers of transmembrane helices were predicted using SignalP [46] and TMHMM [47] respectively. ORFans were identified if their BLASTP *E*-value was lower than 1e^-03^ for alignment length greater than 80 amino acids. If alignment lengths were smaller than 80 amino acids, we used an *E*-value of 1e^-05^. Such parameter thresholds have already been used in previous works to define ORFans. Ortholog sets composed of one gene from each of the four genomes *H. massiliensis* strain AP2^T^, *Holdemania filiformis* strain ATCC 51649 (GenBank accession number ACCF00000000), *Solobacterium moorei* strain F0204 (AECQ00000000), and *Erysipelothrix rhusiopathiae* strain Fujisawa (AP012027) were identified using Proteinortho software (version 1.4) [48] by using cut-off values of 30% protein identity and an *E*-value of 1e^-05^. The average percentages of nucleotide sequence identity between corresponding orthologous sets were determined using the Needleman-Wunsch algorithm global alignment technique. Artemis [49] was used for data management and DNA Plotter [50] was used for visualization of genomic features. The Mauve alignment tool was used for multiple genomic sequence alignment and visualization [51].

## Genome properties

**Figure 6:**
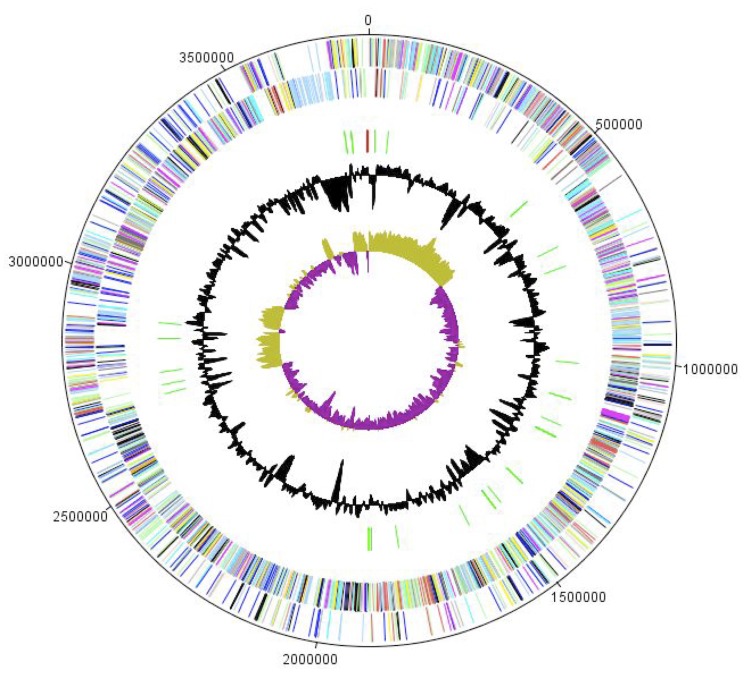
Graphical circular map of the chromosome. From the outside in, the outer two circles show open reading frames oriented in the forward (colored by COG categories) and reverse (colored by COG categories) directions, respectively. The third circle marks the rRNA gene operon (red) and tRNA genes (green). The fourth circle shows the G+C% content plot. The innermost circle shows GC skew, with purple indicating negative values and olive positive values.

The genome of *H. massiliensis* strain AP2^T^ is 3,795,625 bp long (1 chromosome, no plasmids) with a 47.1% G + C content (Figure 6 and Table 4). Of the 3,510 predicted genes, 3,461 were protein-coding genes, and 49 were RNAs. Three rRNA genes (one 16S rRNA, one 23S rRNA and one 5S rRNA) and 46 predicted tRNA genes were identified in the genome. A total of 2,581 genes (74.57%) were assigned a putative function. Two hundred thirteen genes were identified as ORFans (6.06%). The remaining genes were annotated as hypothetical proteins. The properties and the statistics of the genome are summarized in Table 4 and Table 5. The distribution of genes into COGs functional categories is presented in Table 5.

**Table 4:** Nucleotide content and gene count levels of the genome

**Attribute**	Value	% of total^a^
Genome size (bp)	3,795,625	
DNA coding region (bp)	3,333,369	87.82
DNA G+C content (bp)	1,787,739	47.10
Number of replicons	1	
Extrachromosomal elements	0	
Total genes	3,510	100
RNA genes	49	1.39
rRNA operons	1	
Protein-coding genes	3,461	98.60
Genes with function prediction	2,652	75.56
Genes assigned to COGs	2,581	74.57
Genes with peptide signals	258	7.35
Genes with transmembrane helices	908	36.17
CRISPR repeats	7	

^a^ The total is based on either the size of the genome in base pairs or the total number of protein coding genes in the annotated genome

**Table 5:** Number of genes associated with the 25 general COG functional categories

**Code**	**Value**	**%age**^a^	**Description**
J	153	4.42	Translation
A	0	0	RNA processing and modification
K	365	10.55	Transcription
L	137	3.96	Replication, recombination and repair
B	0	0	Chromatin structure and dynamics
D	36	1.04	Cell cycle control, mitosis and meiosis
Y	0	0	Nuclear structure
V	110	3.18	Defense mechanisms
T	165	4.77	Signal transduction mechanisms
M	171	4.94	Cell wall/membrane biogenesis
N	11	0.32	Cell motility
Z	1	0.03	Cytoskeleton
W	0	0	Extracellular structures
U	27	0.78	Intracellular trafficking and secretion
O	56	1.62	Posttranslational modification, protein turnover, chaperones
C	149	4.31	Energy production and conversion
G	372	10.75	Carbohydrate transport and metabolism
E	178	5.14	Amino acid transport and metabolism
F	71	2.05	Nucleotide transport and metabolism
H	43	1.24	Coenzyme transport and metabolism
I	41	1.18	Lipid transport and metabolism
P	119	3.44	Inorganic ion transport and metabolism
Q	44	1.27	Secondary metabolites biosynthesis, transport and catabolism
R	403	11.64	General function prediction only
S	203	5.87	Function unknown
-	880	25.43	Not in COGs

^a^ The total is based on the total number of protein coding genes in the annotated genome.

## Genome comparison with *Holdemania filiformis*, *Solobacterium moorei* and *Erysipelothrix rhusiopathiae*

Here, we compared the genome of *H. massiliensis* strain AP2^T^, with those of *H. filiformis* strain ATCC 51649 (GenBank accession number ACCF00000000), *S. moorei* strain F0204 (AECQ00000000), and *E. rhusiopathiae* strain Fujisawa (AP012027).

The draft genome of *H. massiliensis* is comparable in size to that of *H. filiformis* (3.79 and 3.80 Mb, respectively) and larger in size than those of *S. moorei* and *E. rhusiopathiae* (2.01 and 1.79 Mb, respectively). The G+C content of *H. massiliensis* is smaller than that of *H. filiformis* (47.10 and 50.18%, respectively) but higher than those of *S. moorei* and *E. rhusiopathiae* (36.80 and 36.60%, respectively).

**Figure 7:**
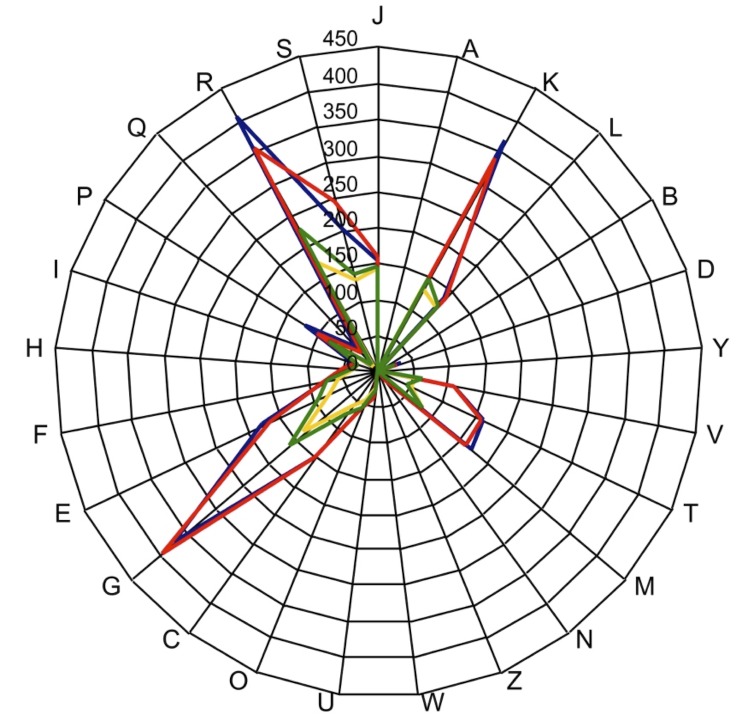
Distribution of functional classes of predicted genes on *Holdemania massiliensis* (colored in blue), *Holdemania filiformis* (colored in red), *Solobacterium moorei* (colored in green) and *Erysipelothrix rhusiopathiae* (colored in yellow) chromosomes according to the clusters of orthologous groups of proteins.

The gene content of *H. massiliensis* is lower than that of *H. filiformis* (3,510 and 4,272, respectively) but higher than those of *S. moorei* and *E. rhusiopathiae* (2,081 and 1,780, respectively). The ratio of genes per Mb of *H. massiliensis* is smaller to those of *H. filiformis*, *S. moorei* and *E. rhusiopathiae* (926, 1,124, 1,035 and 994, respectively). However, the distribution of genes into COG categories was almost similar in all compared genomes (Figure 7).

The nucleotide sequence identity ranged from 62.02 to 84.32% among compared genomes. Table 6 summarizes the numbers of orthologous genes and the average percentage of nucleotide sequence identity between the different genomes studied.

**Table 6:** The numbers of orthologous proteins shared between genomes (upper right)^*^

	*H. massiliensis*	*H. filiformis*	*S. morei*	*E. rhusiopathiae*
*H. massiliensis*	**3,461**	1,005	870	690
*H. filiformis*	84.32	**4,223**	866	686
*S. morei*	63.82	62.88	**1,982**	551
*E. rhusiopathiae*	63.14	62.02	65.48	**1,697**

^*^average percentage similarity of nucleotides corresponding to orthologous proteins shared between genomes (lower left) and the numbers of proteins per genome (bold).

## Conclusion

On the basis of phenotypic, phylogenetic and genomic analyses, we formally propose the creation of *Holdemania massiliensis* sp. nov. that contains the strain AP2^T^. This bacterial strain has been found in Marseille, France.

### Description of *Holdemania massiliensis* sp. nov.

*Holdemania massiliensis* (mas.si.li.en′sis. L. masc. adj. massiliensis of Massilia, the Roman name of Marseille, France, where the type strain was isolated).

Colonies were 0.2 mm in diameter on blood-enriched Columbia agar and Brain Heart Infusion agar. Cells are rod-shaped with a mean diameter of 0.57 µm and a mean length of 1.75 µm. Strictly anaerobic. Growth occurs between 25 and 45°C, with optimal growth observed at 37°C. Cells stain Gram-positive, and are non-motile. Cells are positive for β-galactosidase, α-glucosidase, α-fucosidase and pyroglutamic acid arylamidase. Positive reactions were observed for glycerol, D-ribose, D-galactose, D-glucose, D-fructose, D-mannose, inositol, D-mannitol, D-sorbitol, N-acetyl-glucosamine, amygdalin, arbutin, esculin, salicin, D-cellobiose, D-maltose, D-lactose, D-saccharose, D-melezitose, gentiobiose, D-tagatose and potassium gluconate. Cells are susceptible to amoxicillin, metronidazole, vancomycin, clindamycin and imipenem. The G+C content of the genome is 47.10%. The 16S rRNA and genome sequences are deposited in GenBank under accession numbers JX101683 and CALK00000000, respectively.

The type strain AP2^T^ (= CSUR P195 = DSM 26143) was isolated from the fecal flora of French Caucasian female suffering from severe restrictive anorexia nervosa [3].
